# Educational Case: Mycosis fungoides

**DOI:** 10.1016/j.acpath.2026.100291

**Published:** 2026-08-17

**Authors:** Aneri B. Patel, Janellen Smith, Bonnie Lee, Kristin Olson

**Affiliations:** a University of California, Department of Dermatology, Irvine, USA; b University of California, School of Medicine, Davis, USA; c University of California, Department of Pathology and Laboratory Medicine, Davis, USA

**Keywords:** Pathology competencies, Organ system pathology, Skin, Skin neoplasia, Cutaneous t-cell lymphoma, Mycosis fungoides, Histology, Clinical presentation, Diseases of the skin

The following fictional case is intended as a learning tool within the Pathology Competencies for Medical Education (PCME), a set of national standards for teaching pathology. These are divided into three basic competencies: Disease Mechanisms and Processes, Organ System Pathology, and Diagnostic Medicine and Therapeutic Pathology. For additional information, and a full list of learning objectives for all three competencies, see https://doi.org/10.1016/j.acpath.2023.100086.^1^

## Primary objective

Objective SK5.5: Cutaneous T-cell Lymphoma. Describe the various clinical presentations of cutaneous T-cell lymphoma, specifically mycosis fungoides, and discuss the natural course of the disease.

Competency 2: Organ System Pathology; Topic: Skin (SK); Learning Goal 5: Skin Neoplasia.

## Secondary objective

Objective SK1.1: Pathophysiology of Changes in the Skin. Describe the pathophysiologic basis for changes in the color, surface texture, swelling, temperature, and sensitivity of skin.

Competency 2: Organ System Pathology; Topic: Skin (SK); Learning Goal 1: Classification of Skin Disease.

## Patient presentation

A 66-year-old woman presents to her dermatologist with a pruritic, erythematous, diffuse rash across her body, manifesting as scaly plaques over her arms, chest, back, and lower extremities. Initially, the eruption was only present on her lower legs. Its eventual spread to her back and arms prompted her to seek medical care. This rash has been present and gradually worsening over a period of 3 to 4 years.

She uses Aquaphor and 12% ammonium lactate lotion on the rash twice daily, which provides partial relief from the pruritus. The patient denies any fever, chills, headache, joint pain, or night sweats. She has a history of recurrent urinary tract infections, for which she takes nitrofurantoin. Over-the-counter medications include amoxicillin (for a previous ear infection), ibuprofen, and diclofenac gel applied to the forehead to relieve headaches. She endorses a mild cough that has persisted for over the past 10 years and is worsened by the winter season. Additionally, she is a former cigarette smoker with a 30-pack-year history, having quit 15 years ago. She has two pet cats at home. She has had an unintentional 10-pound weight loss over the years. Her travel history includes a visit to Peru and South Africa approximately 4 years ago.

## Diagnostic findings, Part 1

**Fig. 1:**
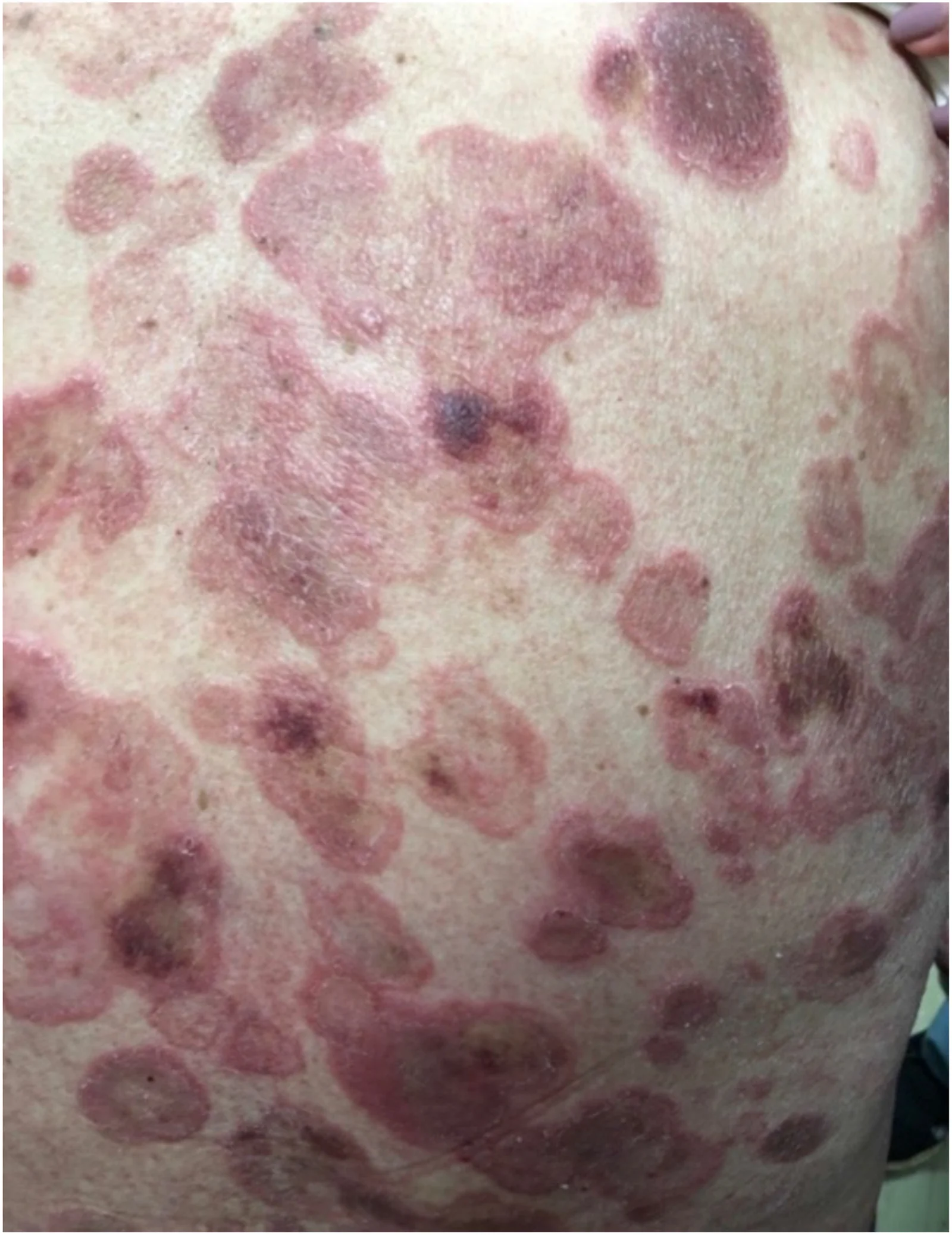
Numerous ill-defined red plaques present on the back.

**Fig. 2:**
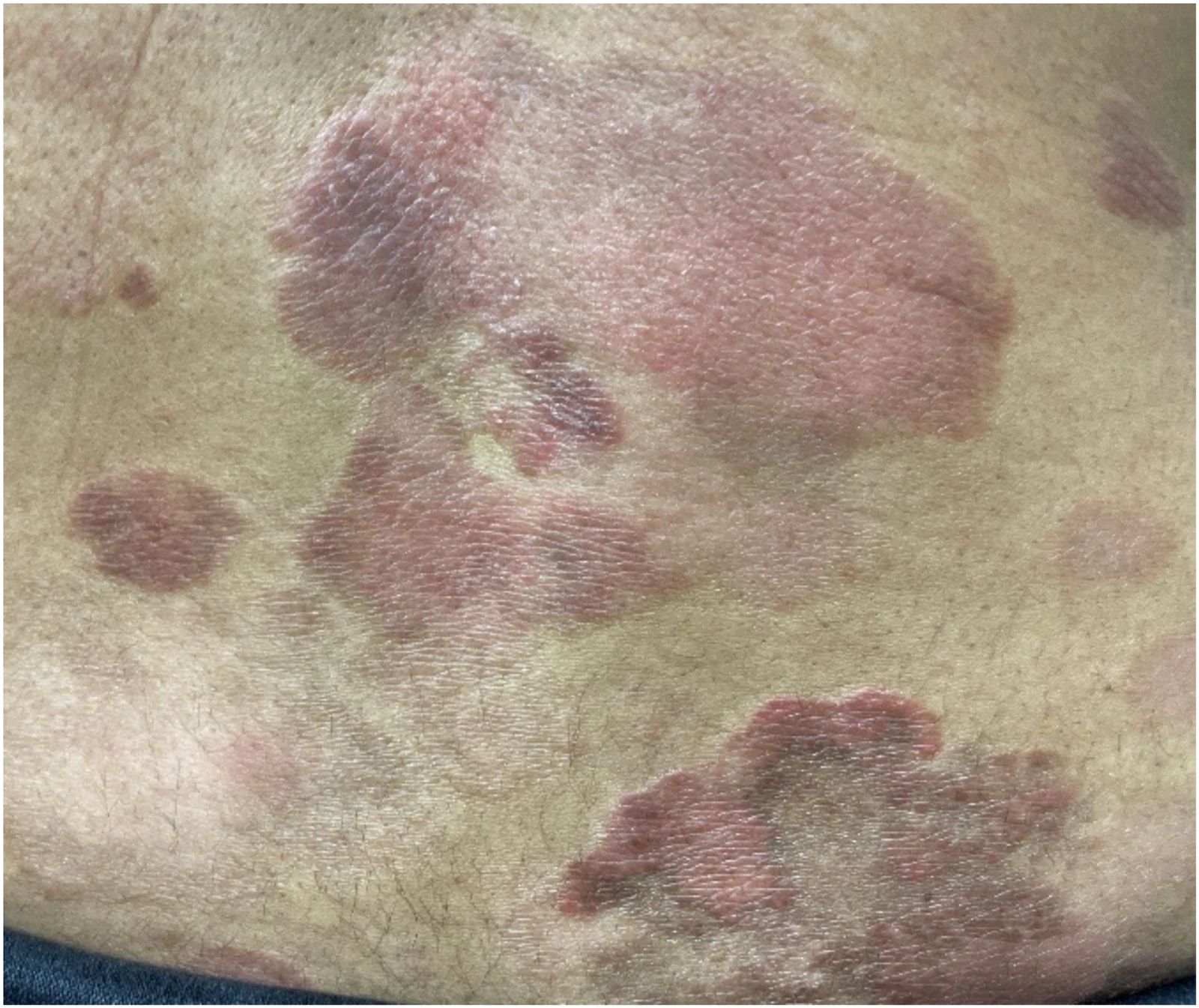
A close up of multiple, irregular, well-demarcated pink-to-violaceous patches and plaques on the skin with fine surface scale**,** surrounded by relatively normal-appearing skin.

On physical examination, the patient is afebrile and normotensive. Inspection of the skin shows numerous scattered ill-defined pink-red plaques across her body, especially over the back, bilateral thighs, and lower legs, please see Fig. 1, Fig. 2. Some of these patches are associated with telangiectatic vessels and some are slightly elevated. These lesions are nontender and without fluctuance or drainage. She also has a poikilodermatous, violaceous eruption across her breasts. There are no significant findings in her armpits or groin, and no lesions are present on her scalp, hands, or feet. Palpation reveals no lymphadenopathy, and the rest of the physical examination is unremarkable.

## Questions/discussion points, Part 1

### Given the clinical presentation, what is the differential diagnosis?

The patient has diffuse nontender erythematous plaques. Clinically, the differential diagnosis includes psoriasis, eczema/contact dermatitis, lichen planus, drug eruption, and cutaneous T-cell lymphoma (CTCL) and its subtypes (e.g. Sezary syndrome [SS] and mycosis fungoides [MF]).

Psoriasis often presents with well-demarcated, scaly plaques over the extensor surfaces of the body. The lack of the typical micaceous scale and involvement of less characteristic areas makes psoriasis unlikely in this patient. Eczema or contact dermatitis usually presents as localized dry, scaly, pruritic circular/oval-shaped erythematous or pink plaques triggered by the presence of environmental allergens (e.g. pollen, dust mites, or mold) or irritant substances (e.g. soaps and detergents). Our patient has persistent, widespread disease without an identifiable trigger, which renders eczema less likely. Lichen planus is characterized by inflamed, raised, bumpy red and purple papules that are most commonly idiopathic but may be associated with infection, medication or trauma. These are difficult to distinguish clinically from other similar entities in the differential diagnosis, often requiring histopathological correlation to confirm the diagnosis. Drug eruption or drug-induced dermatitis can be a maculopapular erythematous to violaceous symmetric eruption that can occur in response to certain medications, including amoxicillin, ibuprofen and nitrofurantoin, with the potential for recurrence at the same site if these drugs are reintroduced. Given that the patient's rash is asymmetric, widespread and lacking a clear correlation with these medications, this diagnosis is less likely. CTCL has multiple subtypes including SS, which presents with erythroderma, lymphadenopathy and systemic symptoms. Our patient mentions a 10-pound unintentional weight loss over 3–4 years, which potentially fits with a slow-growing malignancy, but she lacks erythroderma or lymphadenopathy. Mycosis fungoides (MF) is another subtype of CTCL with a chronic indolent course and lesions that often appear in sun-protected areas, such as the back, buttocks and inner thighs. These lesions appear as thickened red, pink or purple plaques that can be pruritic, with an appearance similar to that of the rash in this patient.

### What diagnostic studies would be the most appropriate to help narrow down the differential diagnosis?

A shave biopsy of one of the patient's most active lesions is obtained. Ideal biopsy locations include regions with more “activity,” such as those that are growing, developing a redder or duskier color, or becoming inflamed or scaly. Skin biopsy is the gold standard for diagnosis and remains a critical step in differentiating between conditions that can appear similar to the naked eye, such as psoriasis, eczema, drug-induced dermatitis, and MF. A shave biopsy is minimally invasive yet usually adequate for histopathologic examination. Given the superficial nature of patch- and most plaque-type lesions, a deeper punch biopsy does not always add significant value.

Hematoxylin and eosin (H&E) staining is the standard first step for nearly all biopsies because it shows overall tissue architecture and cellular morphology, which often helps narrow down the diagnosis. Immunohistochemistry (IHC) is an adjunct test that uses antibodies to label specific proteins in the tissue, helping identify the cell types involved and their abnormal patterns. IHC is especially useful when H&E findings are subtle or overlapping, such as in suspected MF, because it can support or confirm a specific disease process. In addition, molecular testing, most commonly polymerase chain reaction (PCR) based T-cell receptor (TCR) gene rearrangement studies (or sequencing-based clonality assays) can provide evidence of a clonal T-cell population and is an important component of the diagnostic workup for MF, particularly when clinicopathologic findings are equivocal.

Along with the physical examination and biopsy with these studies, a complete blood count, comprehensive metabolic panel, lactate dehydrogenase level, and flow cytometry of the peripheral blood can be of benefit in establishing the diagnosis and developing a treatment plan.

## Diagnostic findings, Part 2

**Fig. 3:**
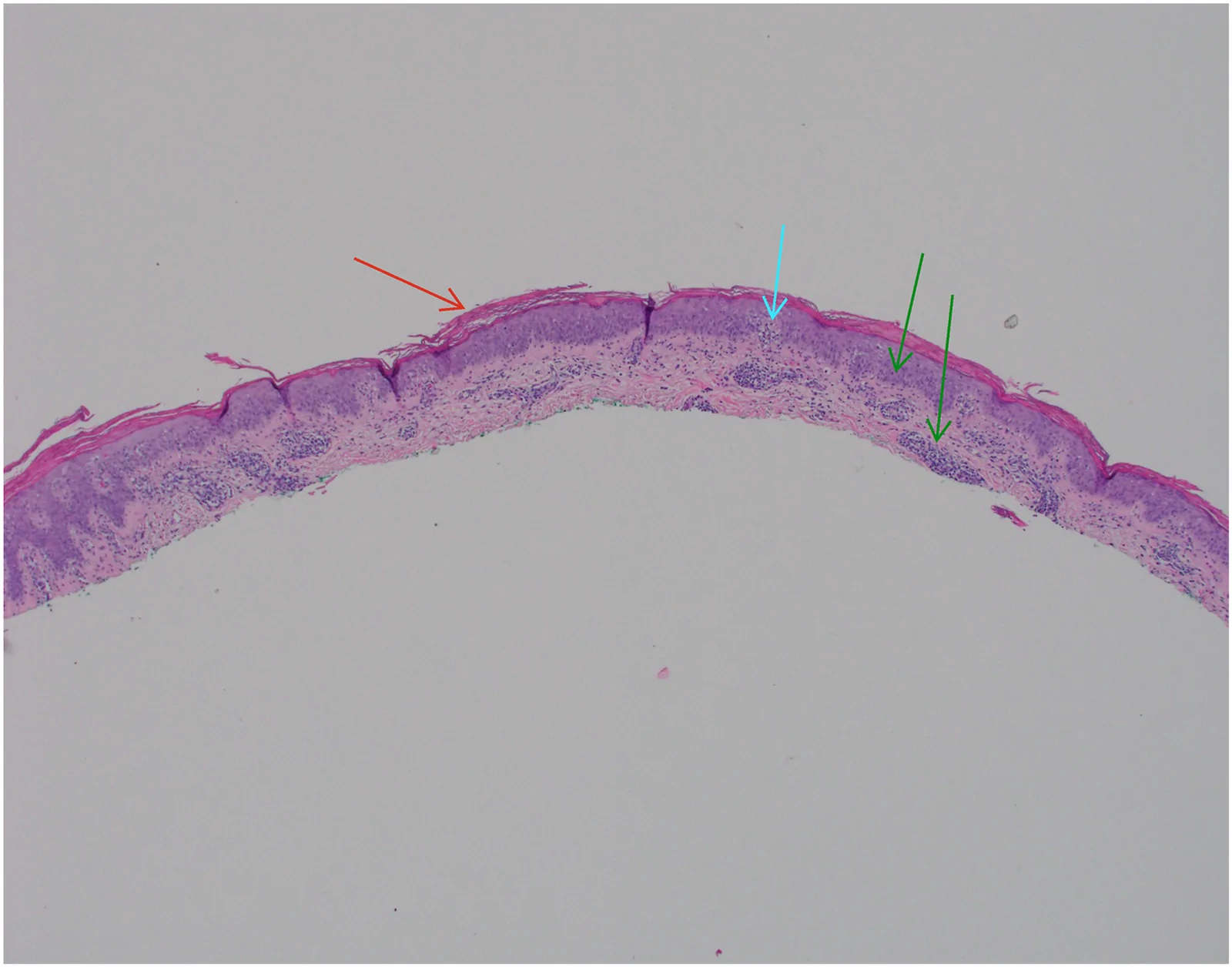
Hematoxylin and eosin (H&E) stain of the lesional biopsy (40x magnification). Slight spongiosis (blue arrow) and hyperkeratosis (red arrow) are visible, and there is increased cellularity in the epidermis and dermis (green arrows).

**Fig. 4:**
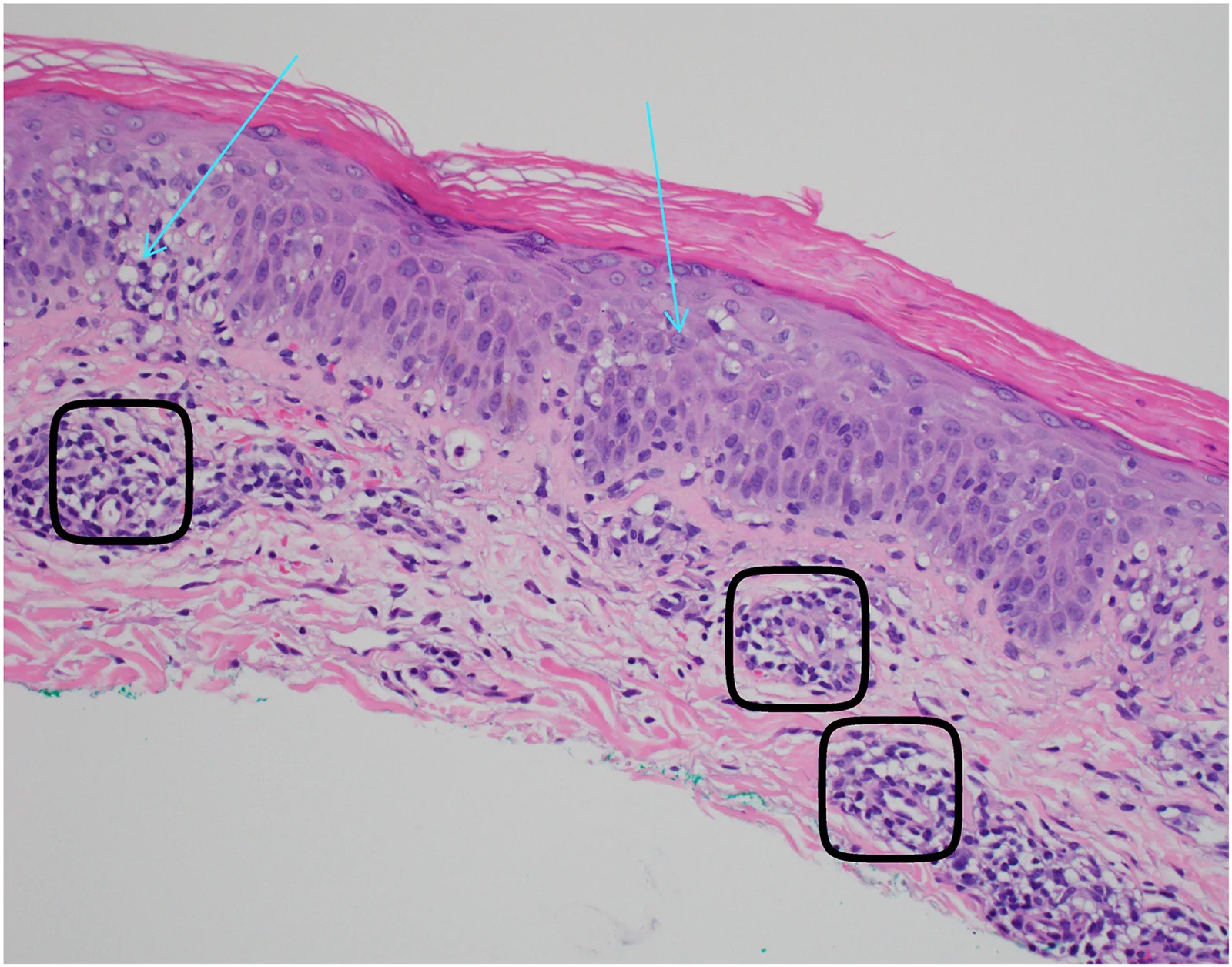
H&E stain of the lesional biopsy (200x magnification) shows small-to medium-sized, irregularly shaped lymphocytes in the epidermis (blue arrows). The papillary dermis shows a patchy perivascular infiltrate of irregularly shaped lymphocytes (black boxes). H&E: hematoxylin and eosin.

**Fig. 5:**
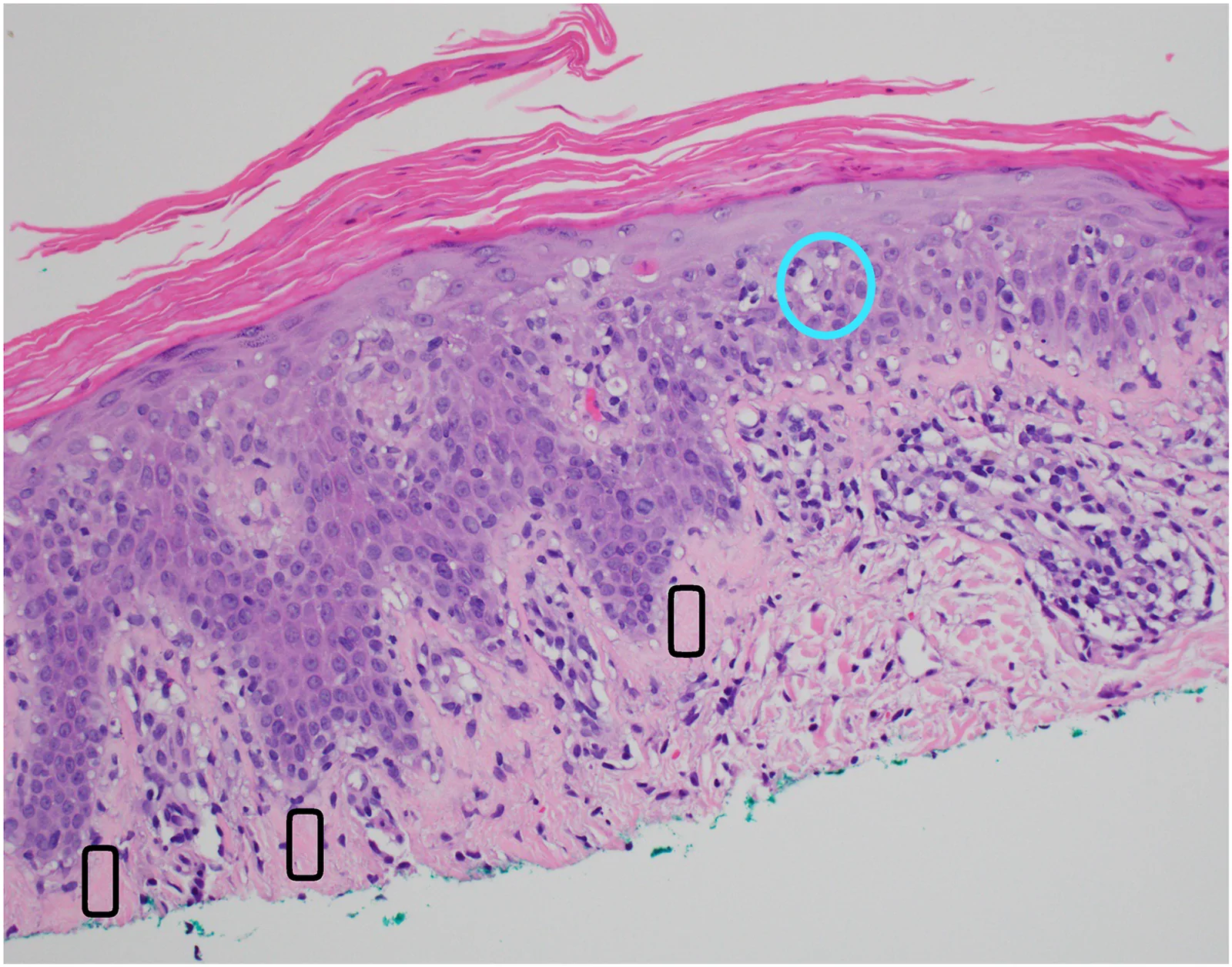
H&E stain of the lesional biopsy (200x magnification) shows lymphocytes lining up along the basement membrane and aggregates of lymphocytes within the epidermis forming small “Pautrier microabscesses” (blue circle). The papillary dermis shows fibrosis (black boxes) and a patchy lymphocytic infiltrate. H&E: hematoxylin and eosin.

Shave biopsies are obtained and shown in Fig. 3, Fig. 4, Fig. 5.

## Questions/discussion points, Part 2

***Evaluate the histopathological findings from*** Figs. 3, Fig. 4, Fig. 5, ***which shows the hematoxylin and eosin (H&E) stain results, and provide the most likely diagnosis***.

**Fig. 6:**
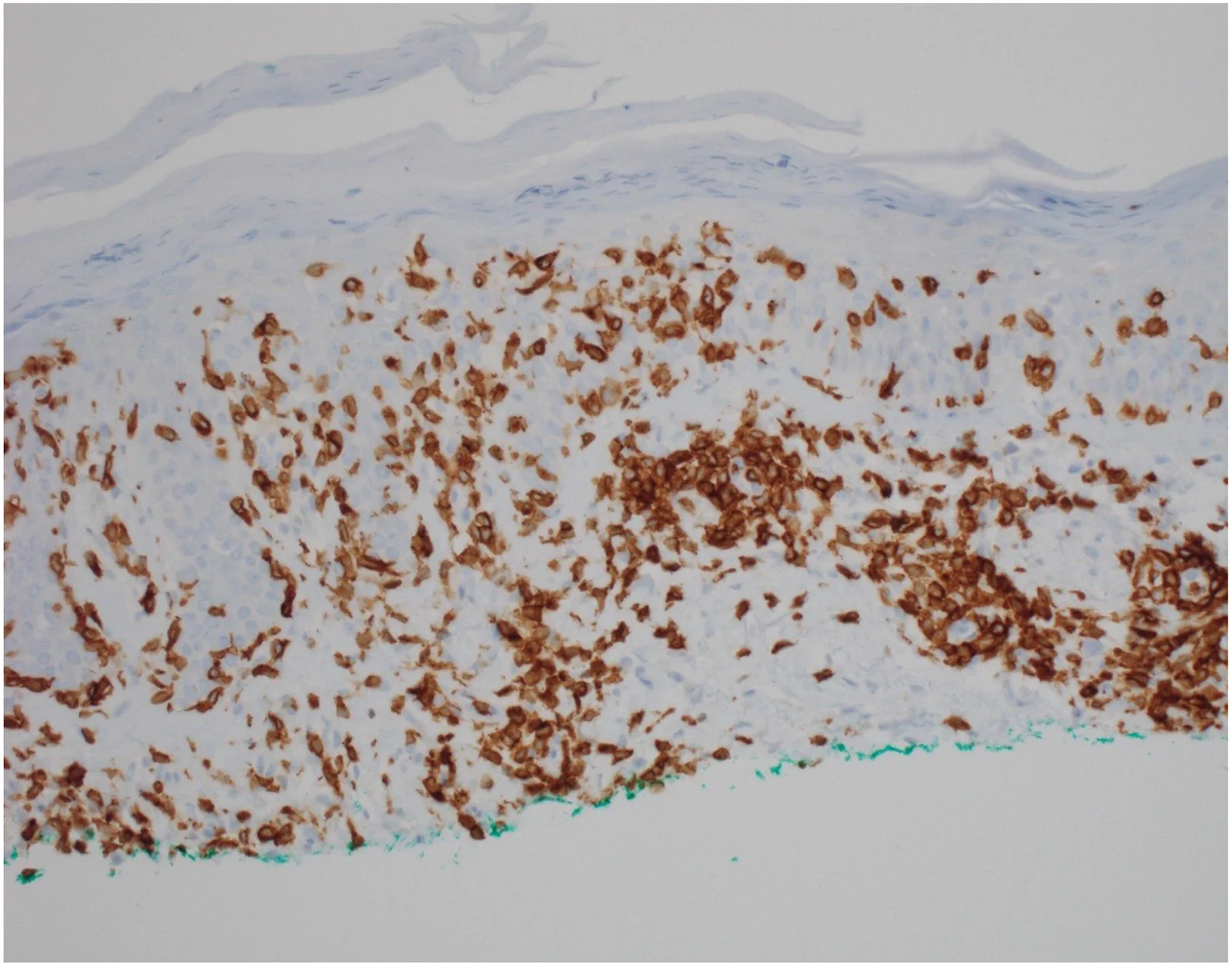
Lesional biopsy (200x magnification). CD4 immunohistochemical (IHC) stain shows increased distribution and density of CD4^+^ lymphocytes especially in the epidermis (depicted by brown coloration).

On H&E sections, lesional skin demonstrates epidermotropism by atypical small-to-medium-sized lymphocytes with irregular nuclear contours (see Fig. 6). Foci of lymphocytes align along the basement membrane, with small intraepidermal aggregates consistent with Pautrier microabscesses. The superficial dermis shows a patchy lymphocytic infiltrate with papillary dermal fibrosis. Taken together, these findings are most consistent with MF**.**

**Fig. 7:**
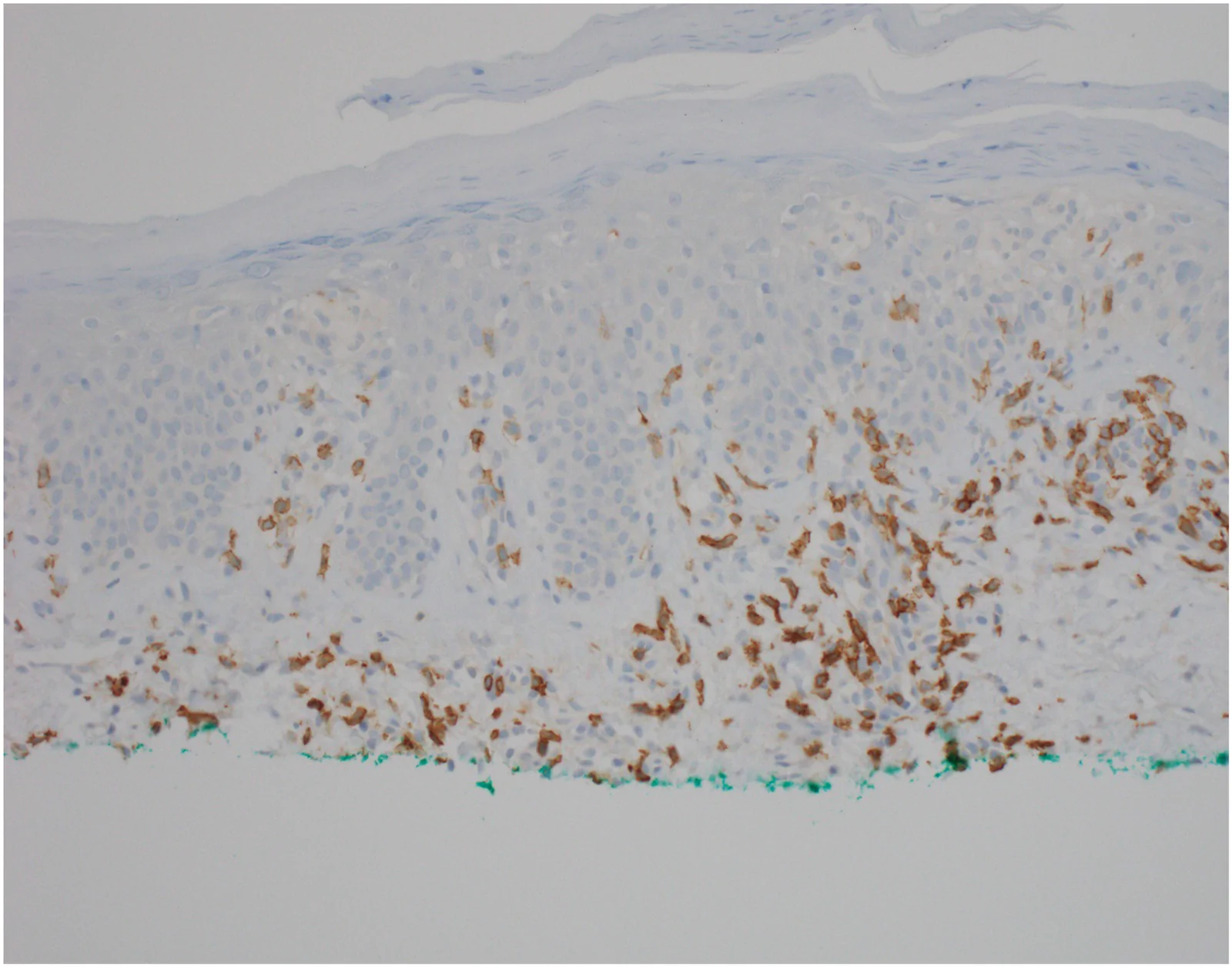
Lesional biopsy (20x magnification). CD8 immunohistochemical stain shows the epidermis with rare reactive lymphocytic inflammation; however, the majority of the intraepidermal lymphocytes are CD8^−^.

**Fig. 8:**
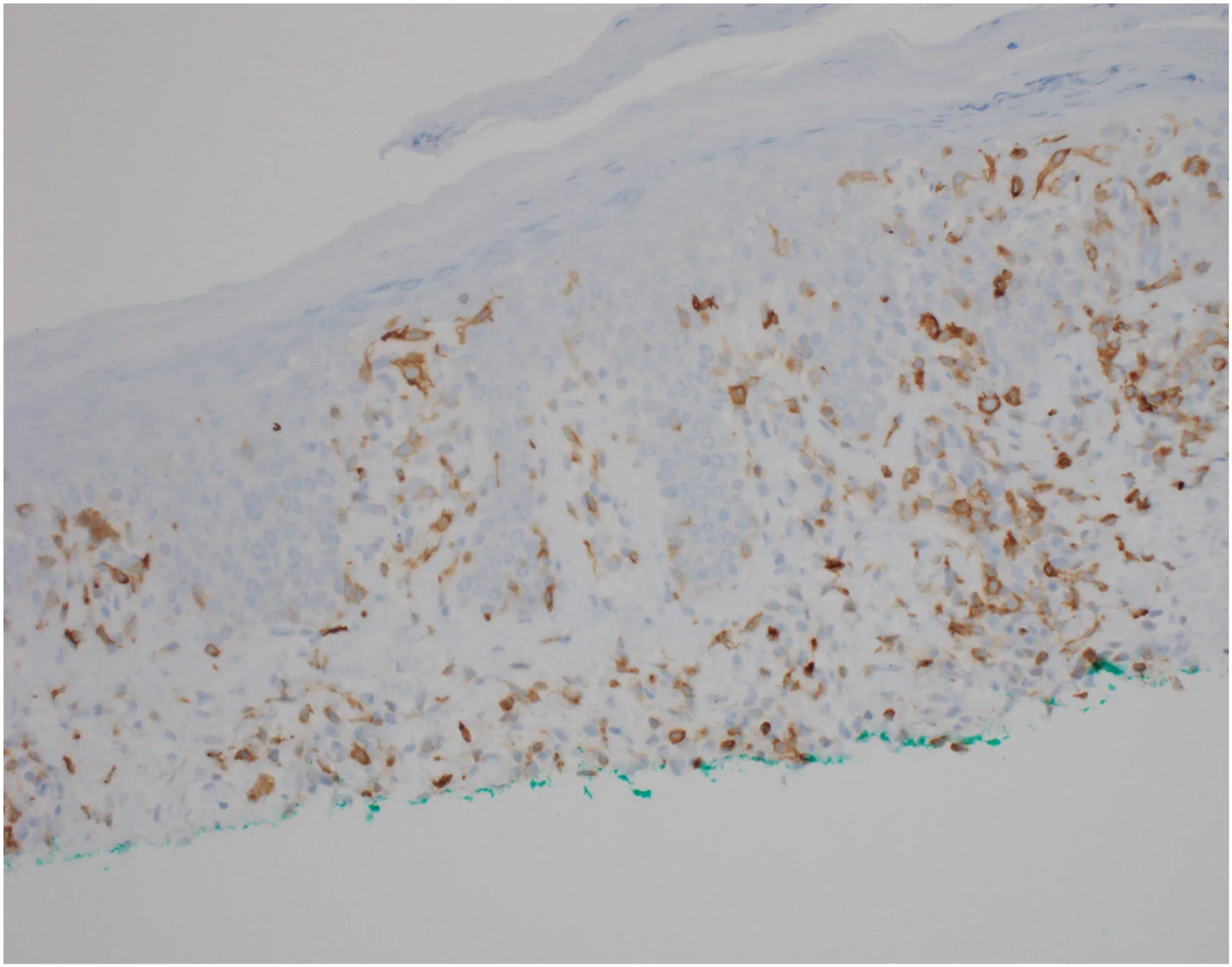
IHC stain for CD7 (20x magnification) shows a few reactive cells in the epidermis; however, there is overall diminished expression of CD7.

***Describe the immunohistochemical (IHC) stain findings from*** Figs. 6, Fig. 7, Fig. 8.

The cluster of differentiation (CD) markers demonstrated in the immunohistochemical stains above are used to identify specific cell-surface proteins expressed by leukocytes. Therefore, these stains would be helpful in the diagnosis of CTCL and MF. Normal T cells express CD3, CD4 (helper T cells), CD8 (cytotoxic T cells), CD5, CD7, CD2, and CD26 (memory cells and mature T-cells) as these proteins are critical for T-cell function and signaling. Normal T cells typically lack CD20 (B-cell marker), CD30 (expressed only in activated T cells), and CD56 (NK cell marker). In CTCL, malignant T cells frequently show loss of CD2, CD5, CD7 and CD8 while retaining CD3 and CD4, a pattern that helps distinguish them from normal reactive T cells.^2^^,^^3^ Therefore, the IHC results shown in the figures above further supports the diagnosis of MF, a subtype of CTCL (Table 1^2^, ^3^, ^4^).

**Table 1:** Summary of histopathological findings in MF and SS.^2^, ^3^, ^4^

Feature	Normal skin	Mycosis fungoides (MF)	Sezary syndrome (SS)
Overall microscopic pattern	No atypical lymphoid infiltrate; may see only scattered, mild perivascular inflammation in the superficial dermis.	Superficial dermis shows a patchy to band-like infiltrate of atypical lymphocytes, often with epidermal involvement.	Findings can be subtle or nonspecific; may resemble chronic eczematous/erythrodermic dermatitis with a superficial perivascular lymphoid infiltrate.
Epidermal involvement (epidermotropism)	Typically, absent.	Common: Epidermotropism with clonal T cells in the epidermis in most cases.	Variable: Epidermotropism may be limited or not seen, even when blood disease is present.
Immunophenotype/marker profile	N/A	Neoplastic T cells are usually CD3^+^ and most often CD4-predominant (CD8-predominant cases occur). Atypical cells frequently show loss of one or more pan-T-cell markers, most often CD7**,** and sometimes CD2 and/or CD5; decreased/absent CD26 may also be observed.	Peripheral blood correlation is key: circulating atypical T cells typically show an aberrant CD4^+^ phenotype, often with an elevated CD4:CD8 ratio, and may demonstrate loss of markers such as CD7 and/or CD26 (with circulating atypical cells).
Cytology of atypical lymphocytes	If lymphocytes are present, they are small, mature-appearing cells without atypia.	Predominantly small-to-intermediate atypical lymphocytes; nuclei may be irregular with hyper convoluted (“cerebriform”) contours.	Similar atypical (“Sézary”) cells may be identified, classically with cerebriform nuclei; tissue changes can be less striking than in classic MF.
Pautrier microabscesses	Not seen.	May be present: Small intraepidermal collections of atypical lymphocytes (not required for diagnosis).	Generally infrequent**,** and when present may be less prominent than in classic MF.

### Correlate the pathophysiologic basis of mycosis fungoides with this patient's clinical findings (patches/plaques, poikiloderma, and telangiectasias). Why do these skin changes occur?

Mycosis fungoides is a CTCL composed of malignant, skin-homing T lymphocytes that preferentially traffic to and persist within the epidermis and superficial dermis. Clinically, erythema is driven largely by the superficial perivascular inflammatory infiltrate and associated vascular dilation, while the patch-to-plaque morphology is multifactorial, reflecting not only the density of the dermal infiltrate but also epidermal changes induced over time, including epidermal hyperplasia (acanthosis) and parakeratosis, which contribute to thickening and scale; epidermotropism and small intraepidermal aggregates (Pautrier microabscesses) remain supportive histopathologic findings. With chronicity, repetitive injury at the dermo-epidermal junction can lead to remodeling of the papillary dermis, including fibrosis and variable epidermal atrophy. These cumulative changes may produce a poikilodermatous appearance (dyspigmentation, atrophy, and telangiectasias), although poikiloderma is not universal and is often most apparent in more indolent, pauci-inflammatory disease when it is not masked by a markedly thickened epidermis or dense infiltrate.

### What are cutaneous lymphomas?

Cutaneous lymphomas are T- or B-cell derived. CTCL is a T-cell lymphoma that initially presents in the skin, distinguishing it from systemic lymphomas with secondary skin involvement. T-cell lymphomas are the most common subtype of cutaneous lymphomas. Cutaneous T-cell lymphomas are a subset of non-Hodgkin lymphomas that include MF, Sezary Syndrome, CD30^+^ lymphoproliferative disorders (such as lymphomatoid papulosis and primary cutaneous anaplastic large cell lymphoma), and erythrodermic T-cell lymphomas).^5^

### What does the term “mycosis fungoides” mean, and what is its pathogenesis?

“Mycosis fungoides” is a misleading moniker, as it suggests a disease caused by a fungus. The term was coined by the famous French dermatologist Baron Jean-Louis Alibert (1768–1837) because the skin lesions that develop in advanced disease have an exophytic, mushroom-like appearance.^4^

MF is characterized by a clonal proliferation of T cells, most commonly CD4^+^ cells that often lack expression of typical T-cell antigens such as CD2, CD5, or CD7. As the clonal T cells accumulate in the dermis, they cluster around Langerhans cells, forming structures known as Pautrier microabscesses. Some malignant T-cells migrate to nearby lymph nodes and eventually enter the bloodstream via lymph, circulating alongside normal T cells.^6^^,^^7^

### What are the microscopic features of the stages of mycosis fungoides?

**Fig. 9:**
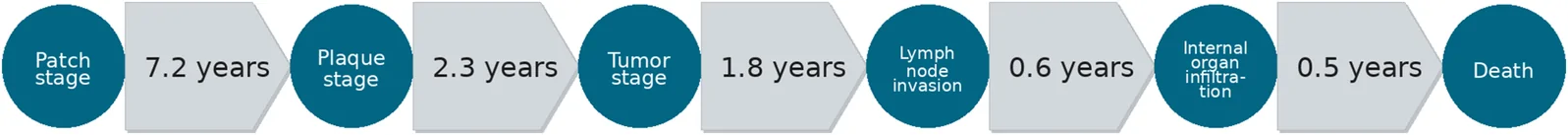
Progression timeline in mycosis fungoides.

There are three stages of MF: the patch stage, plaque stage and tumor stage. The majority of the patients remain in early-stage MF without progression of their disease, while about 8–20% of diagnosed patients progress to more advanced stages.^4^ A recent study showed that, in patients with progressive MF, the disease typically progresses through this sequence of stages, followed by lymph node involvement and internal-organ infiltration, ultimately leading to a fatal outcome. Please take a look at Fig. 9 below, which also references the average time spent at each stage.^8^ Overall, the average disease duration in patients with progression is approximately 12.4 years.^8^

1. Patch stage
  a. This is the earliest stage of MF, and the lesions vary in diameter and appear as erythematous or brownish scaly patches. They can appear in a single location or in multiple locations such as the breasts, gluteal region, and inner thighs.^9^^,^^10^
  b. Microscopic features of early MF are often nonspecific and can overlap with various inflammatory or non-neoplastic skin conditions. This results in a greater time to diagnosis for these patients, which can sometimes be years. The use of topical corticosteroids as part of empirical treatment can also further obscure histologic findings, as steroids reduce inflammation and cause tissue atrophy. This is especially important in MF given that the clonal T cells and characteristic changes in the epidermis and dermis provide diagnostic clarity.
  c. In the late patch stage, with more established lesions, the histopathology shows a lymphocytic infiltration at the edges of the basal layer and epidermotropism (presence of lymphocytes in the epidermis), as was present in our patient. These lymphocytes can be small-to medium-sized and can show slightly rounded or cerebriform nuclei.^10^
2. Plaque stage
  a. The lesions appear larger and are either erythematous, scaly patches or slightly raised plaques. The patient may report the appearance of new lesions over time. These lesions are asymmetrically distributed across the body.
  b. On histopathology, the epidermis often demonstrates acanthosis and a psoriasiform pattern, with minimal spongiosis. A dense subepidermal, band-like lymphocytic infiltrate with cerebriform nuclei is present.^8^ Epidermotropism is more pronounced at this stage, and Pautrier microabscesses are seen in about one-third of cases.^10^
3. Tumor stage
  a. These exophytic lesions resemble “mushrooms.” Often, these lesions are not only larger in diameter but also associated with more itching.^11^
  b. Under the microscope, there is diffuse, dense dermal infiltration by medium- to large-sized lymphocytes, often accompanied by a loss of epidermotropism. This phase may include large-cell transformation, defined as transformation of the clonal lymphocytes from small to large (up to 4x the size of a small lymphocyte) in at least a quarter of the population of tumor cells, indicating a more aggressive form of the disease.^12^ The large cells are either CD30^+^ or CD30^−^. CD30^+^ cells portend a worse prognosis.

### Describe the management of mycosis fungoides.

The management of MF depends on the stage. Phototherapy and topical steroids are more commonly used for localized disease and systemic agents are used for advanced stages.^13^

Localized therapy includes:

• Topical steroid creams and ointments, such as clobetasol or betamethasone
• Phototherapy
• Radiation therapy

Systemic agents include:

• Chemotherapy
• Immunotherapy
• Combination of chemotherapy and radiation therapy

### Can mycosis fungoides be cured? What is the prognosis of mycosis fungoides?

There is no known cure for MF. However, the above treatment options can result in long-term remission. The prognosis for MF varies widely depending on individual factors and stage. Patients with limited patch or plaque-stage MF generally have a life expectancy comparable to that of individuals of the same age, sex, and geographic ancestry without the disease. Recent research indicates that the 10-year disease specific survival rates are 97%–98% for those with limited patch-plaque disease (affecting less than 10% of the skin), 83% for those with generalized patch-plaque disease (involving more than 10% of the skin), 42% for individuals with tumor-stage disease, and approximately 20% for those with confirmed lymph node involvement.^13^, ^14^, ^15^

Several factors are associated with worse outcomes, including:

• Widespread disease
• Extracutaneous involvement (systemic disease), most commonly affecting the lymph node, peripheral blood, liver, spleen, lung, bone marrow, gastrointestinal tract, pancreas, and kidney
• Advanced age (over 60 years)^16^
• Presence of large cell transformation
• Elevated lactate dehydrogenase (LDH) levels (513–7639 U/L)^17^
• Thicker plaques or tumors and/or metastasis at the time of diagnosis

Given the importance of early diagnosis, clinicians should maintain a low threshold for considering MF in the differential diagnosis of psoriasis or eczema, particularly with a rash that is not improving with standard therapies. In such cases, early dermatology referral and skin biopsy should be strongly considered to facilitate timely diagnosis and appropriate management.

### What happened to our patient?

The patient's complete blood count (CBC) showed mildly elevated red blood cells (5.17 million per U/L) (reference range 3.8–5.1 U/L), mildly elevated platelets (413 thousand per U/L) (reference range 140–400 thousand per U/L), complete metabolic panel (CMP) within normal limits, and LDH of 125 UL (reference range 120–250 U/L). Flow cytometry of the blood showed no malignant T cells. A positron emission tomography (PET) scan was negative for nodal or extra-nodal disease. The patient was staged using tumor, node, visceral metastasis, blood (TNMB) guidelines^16^ as clinical stage IB, which warrants the use of localized treatment. She is scheduled to begin phototherapy soon and continues to use triamcinolone 0.1% ointment in the interim. Her lesions appear stable in size.

## Teaching points

• Chronic patches and plaques with a widespread distribution require early consideration of mycosis fungoides (MF) to avoid diagnostic delays.
• MF is distinguished from inflammatory dermatoses by epidermotropic atypical T cells with cerebriform nuclei (often CD4^+^ with loss of CD7/CD26) and Pautrier microabscesses; in addition to light microscopy and IHC, TCR gene rearrangement studies can support the diagnosis by demonstrating a clonal T-cell population. The clinical evolution from patch/plaque lesions to poikiloderma in MF reflects epidermotropism of neoplastic skin-homing T cells, with chronic superficial inflammation leading to epidermal atrophy, papillary dermal fibrosis, dyspigmentation, and telangiectasias
• Phototherapy, corticosteroids, and topical chemotherapy are employed for localized disease, and systemic agents are used for more advanced stages.
• Factors such as age, extracutaneous involvement, and large-cell transformation significantly affect prognosis.

## Consent for participation

Informed consent to participate was verbal for our paper.

## Consent for publication

Informed consent for publication was provided by the participant(s) or a legally authorized representative.

## Funding

The article processing fee for this article was funded by an Open Access Award given by the Society of ‘67, which supports the mission of the Association for Academic Pathology to produce the next generation of outstanding investigators and educational scholars in the field of pathology. This award helps to promote the publication of high-quality original scholarship in *Academic Pathology* by authors at an early stage of academic development.

## Declaration of conflicting interest

The authors declare that they have no known competing financial interests or personal relationships that could have appeared to influence the work reported in this paper.
